# Revolutionizing clinical trials: the role of AI in accelerating medical breakthroughs

**DOI:** 10.1097/JS9.0000000000000705

**Published:** 2023-10-06

**Authors:** Hitesh Chopra, Annu, Dong K. Shin, Kavita Munjal, Priyanka, Kuldeep Dhama, Talha B. Emran

**Affiliations:** a Department of Biosciences, Saveetha School of Engineering, Saveetha Institute of Medical and Technical Sciences, Chennai - 602105, Tamil Nadu, India; b Thin Film and Materials Laboratory, School of Mechanical Engineering, Yeungnam University, Gyeongsan 38541, Republic of Korea; c Department of Pharmacy, Amity Institute of Pharmacy, Amity University, Noida, Uttar Pradesh 201303, India; d Department of Veterinary Microbiology, College of Veterinary Science, Guru Angad Dev Veterinary and Animal Sciences University (GADVASU), Rampura Phul, Bathinda, Punjab; e Indian Veterinary Research Institute (IVRI), Izatnagar, Bareilly, Uttar Pradesh; f Department of Pharmacy, BGC Trust University Bangladesh, Chittagong; g Department of Pharmacy, Faculty of Allied Health Sciences, Daffodil International niversity, Dhaka, Bangladesh

**Keywords:** artificial intelligence, application, clinical trial, difficulties

## Abstract

Clinical trials are the essential assessment for safe, reliable, and effective drug development. Data-related limitations, extensive manual efforts, remote patient monitoring, and the complexity of traditional clinical trials on patients drive the application of Artificial Intelligence (AI) in medical and healthcare organisations. For expeditious and streamlined clinical trials, a personalised AI solution is the best utilisation. AI provides broad utility options through structured, standardised, and digitally driven elements in medical research. The clinical trials are a time-consuming process with patient recruitment, enrolment, frequent monitoring, and medical adherence and retention. With an AI-powered tool, the automated data can be generated and managed for the trial lifecycle with all the records of the medical history of the patient as patient-centric AI. AI can intelligently interpret the data, feed downstream systems, and automatically fill out the required analysis report. This article explains how AI has revolutionised innovative ways of collecting data, biosimulation, and early disease diagnosis for clinical trials and overcomes the challenges more precisely through cost and time reduction, improved efficiency, and improved drug development research with less need for rework. The future implications of AI to accelerate clinical trials are important in medical research because of its fast output and overall utility.

## Introduction

Highlights

- Clinical trials are the key evaluation for the development of safe, dependable, and efficient therapeutics.
- By collecting data in a way that enhances recruitment, adherence, and data analysis, artificial intelligence (AI) has the potential to accelerate trial cycles and patient outcomes.
- AI can automatically fill up the necessary analysis report and intelligently interpret the data to feed downstream systems.

Artificial intelligence (AI) is a field of computer science that tries to figure out how the human brain solves problems and makes decisions^1^. It has been around for almost 100 years, and the use of AI is not what makes it new. Over the past few decades, drug development has become more complex, and AI has been used to help with that, though this is not talked about much. A famous example is using AI models to help figure out how the structure of chemical molecules affects how they work in living things^1,2^. They are important for finding new drugs and help scientists predict how a potential drug will work in the body. Even though their estimates are limited by what the models can do, they have made the process of finding new drugs much more efficient by letting scientists focus on possible drugs that have a better chance of fighting a certain disease^3,4^.

The problems we are attempting to tackle now include fighting considerably more complicated illnesses with more precision, safety, and effectiveness than was possible in the past. Fortunately, we live in a time where not only is there a plethora of data on human biology but also the capacity to analyse enormous volumes of that data, all owing to cheap and powerful technology. Although AI’s ability to tackle these complicated illnesses has grown, so has the challenge of doing so.

Using massive volumes of genetic, phenotypic, and chemical data, we can now create a whole virtual world centred on drug development, complete with in-silico models that replicate human illness. Due to their reliance on a single preset hypothesis, traditional illness discovery approaches often miss identifying traits that may be identified using computational methods and algorithms. Multiple targets might be considered at once as we assess therapy options. As human beings, we are incapable of multitasking. AI helps bridge that gap, but it still relies on humans for direction.

In recent years, the coronavirus disease 2019 (COVID-19) outbreak has pushed the pharmaceutical business to go through more digital change^5^. More people are interested in using AI and big data analytics across the pharmaceutical value chain, from drug development and clinical study design all the way to sales and marketing. This is because ultralarge datasets are now available, and technology is getting better. In the past 3–4 years, there has been more interest in using AI to find new drugs. This is shown by the growing number of start-ups working in this area, the growing number of agreements for drug development, and the record amounts of funding. Most drugs made with AI are still in the early stages of development, but there have been some big steps forward recently. For example, the first drug made with AI is now in clinical studies, and a drug already on the market is being used to treat COVID-19.

In the past few years, AI has caught the attention and interest of people who work in medical technology. This is because a number of companies and big research labs have been working to make AI technologies ready for clinical use^6,7^. AI, also known as Deep Learning (DL), Machine Learning (ML), or Artificial Neural Networks (ANNs), can now help doctors in the real world for the first time. These tools could change the way clinicians do their jobs and make them more productive while also improving care and patient turnover. AI for drug discovery is a technology that uses machines to mimic human intelligence in order to solve difficult problems in the process of making new drugs. Adopting AI solutions in the clinical trial process gets rid of possible problems, shortens the time it takes to run a clinical trial, and makes the process more accurate and productive. Life science industry players are becoming more interested in using these advanced AI solutions in the drug development process. In the pharmaceutical industry, it helps find new chemicals, find treatment targets, and make more personalised medicines. AI systems used for drug development can be a good way to learn more about how to find drugs to treat and lessen the effects of a number of chronic diseases. For instance, NVIDIA Corporation released Clara Holoscan MGX in March 2022 so that real-time AI apps could be made and used^8^. Clara Holoscan MGX expands the Clara Holoscan platform to offer an all-in-one, medical-grade standard design and long-term software support to speed up innovation in the medical device market. This will help the company improve the AI it uses for treatment, diagnosis and finding new drugs. In May 2022, BenevolentAI, a top clinical-stage AI-enabled drug discovery business, said that AstraZeneca had chosen an extra novel target for idiopathic pulmonary fibrosis (IPF) for its drug development pipeline^9^. This is the third new target to come out of the partnership. It was found using the Benevolent Platform for IPF and chronic kidney disease (CKD), and then AstraZeneca tested it and chose it for its collection. This builds on the agreement signed in January 2022 to extend the partnership with AstraZeneca to include systemic lupus erythematosus and heart failure as two new disease areas. This has helped the company get along better with each other.

The potential for AI to transform the field of medicine and the way patients are cared for is enormous. AI’s capacity to sift through mountains of data, spot trends, and make precise predictions has the potential to hasten the development of new treatments as well as improve trial design, patient recruitment and selection, safety monitoring, and drug discovery. AI has the potential to bring us closer to personalised medicine and more effective therapies by simplifying procedures, decreasing costs, and enhancing efficiency. We are taking the first steps on a revolutionary path towards a future where scientific progress is expedited, patient outcomes are better, and medical discoveries are available to everybody as we continue to explore the tremendous potential of AI in clinical trials^10–14^.

## Traditional process of clinical trials

### Typical human subjects’ research procedures and schedule

In order to guarantee the safety and effectiveness of new medications, linear and sequential clinical trials serve as the gold standard. Under-enrolment, attrition in the middle of the study, unforeseen side effects, and contradictory data are all factors that contribute to its failure. A clinical trial consists of four stages once the first steps of discovery and preclinical research are complete^15^.

**Figure 1:**
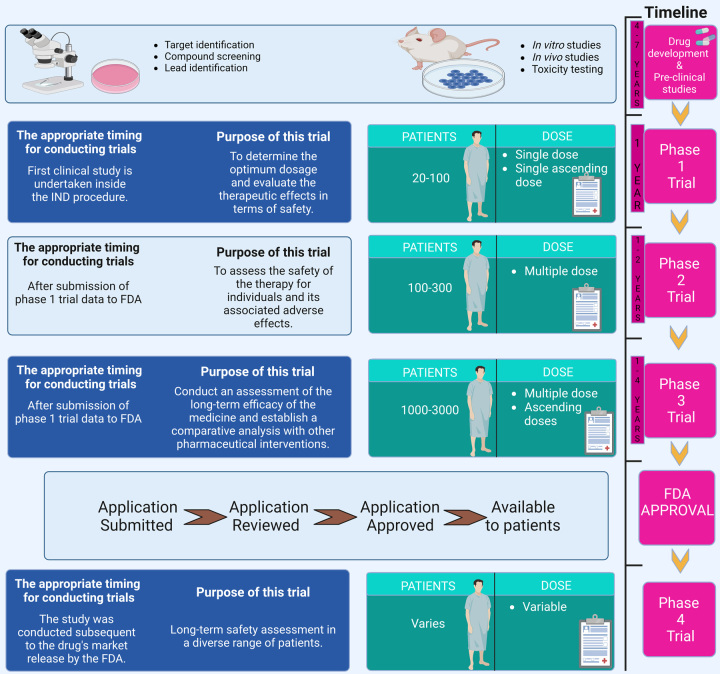
Various steps FDA drug approval for clinical trials. FDA, Food and Drug Administration.

Phase I involves the initial testing of a medicine or therapy on a small number of patients (20–80) to learn about safety and identify negative effects. Approximately 70% of people who start this process progress to the next level within 3–6 months. In phases II and III, the medicine or therapy is tested on a larger sample size (100–300 people) to evaluate its efficacy and further evaluate its safety. After roughly a year or two, around 33% of people go on to the last phase. Phase III involves 10 times as many participants as Phase II (1000–3000) to establish efficacy, find and monitor adverse effects, and compare results to those of other available therapies (as shown in Fig. 1). This may take anywhere from 1 year to 4 years, and only around a quarter to a third of participants make it to the next level. Phase IV occurs after a medicine has been given the green light by the Food and Drug Administration (FDA) and is made accessible to the general population^15^. Scientists are still monitoring its effects on the general population to determine its best applications and ensure its safety. Given the rigorous regulatory scrutiny a medicine or therapy must pass before reaching this stage, it is not surprising that this process might take a year or more and have a success rate of 70–90%. Subtherapeutic doses are tested on a limited number of people (10–15) in the first phase I of certain studies^16,17^.

## The point where hope and reality collide

Due in large part to the normalisation of high clinical trial failure rates over the last several decades, fewer new medications are reaching the market despite rising pharmaceutical R&D expenditures. When it comes to developing complicated novel medicines, particularly ones that are intended for smaller, diverse patient groups, traditional clinical trials lack the analytical sophistication, flexibility, and speed required. High trial failure rates and increasing R&D expenses may be attributed in part to inefficient patient screening and recruitment methods, as well as difficulties in tracking participants’ adherence to treatment protocols. One of the most critical aspects of designing a clinical study is determining the health criteria that will be used to choose participants. Participants are selected from a large pool based on a variety of factors, the most important of which may be whether or not they have the illness or condition being studied. Eligibility may also be determined based on other factors found in a patient’s medical record, such as bilirubin, haemoglobin, platelet counts, blood pressure, red and white blood cell counts, and many more. Patients who do not satisfy the inclusion criteria will be excluded from the research. Setting strict criteria is thought to protect vulnerable patients from the potential side effects of untested drugs; however, researchers have discovered that, depending on the drug and condition being tested, patient withdrawal rates due to adverse side effects can be virtually the same whether a trial used relatively aggressive or weak restrictions^18^. When it comes to preventing the kinds of harm they are trying to avert, many prohibitions are mostly ineffective. Exclusion criteria are also not always uniform among trials and are occasionally determined at random. If studies had used modelling, data aggregation and extrapolation from rich databases to find the optimal participant pool to decrease attrition and unjustified disqualification, many unsuccessful trials may have succeeded. Especially in phase III trials, which are the most complex and expensive, billions of dollars will be wasted if the best-suited patients are not brought to the trial on time with the technical infrastructure to cope with the complexity of running the trial.

A clinical trial’s effectiveness might be jeopardised by participants’ adherence or lack thereof^19,20^. The burden of ensuring that therapy is correctly received and data are adequately gathered typically falls on patients due to the use of inadequate data collection and verification mechanisms for monitoring and coaching. Adherence may be increased, and by extension, healthcare results, with the use of wearable gadgets driven by AI technology. Faxing patient data, physically counting pills, and depending on patients’ diaries to assess medication adherence are all examples of antiquated practises that are all too widespread. Negative health outcomes, higher recruitment expenses, and skewed research results are only some of the potential consequences of poor adherence. However, up to 50% of prescriptions given in the United States are taken wrongly, despite the fact that adherence rates of 80% or higher are essential for therapeutic effectiveness^21,22^.

## AI and clinical trials

The fate of a clinical study may rest on the shoulders of those responsible for its earliest phases. A successful identification of participants will boost the trial’s effectiveness potential; however, patient selection and recruiting may be time-consuming and resource-intensive. The failure of a study may be attributed to inefficient or slow recruiting efforts. This risk of monetary loss should be seen as motivation to use AI technology throughout the earliest phases of clinical trials.

**Figure 2:**
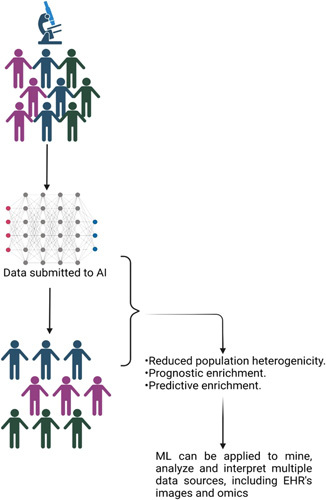
Use of AI in clinical trials. AI, artificial intelligence.

AI can sift through mountains of data to identify subsets of patients who could respond well to a clinical study. Social media material may be analysed to identify hotspots for a disease or disorder, which helps focus the recruitment effort. By assessing hospital medical data and notifying both physicians and patients about clinical trial prospects, AI has the potential to speed up the process of discovering suitable participants. Technology has the ability to streamline formerly complicated admissions requirements, making them more accessible to qualified applicants. A clinical trial’s potential participants may be uncovered by using AI to comb through mountains of medical information. Researchers at New York’s Mount Sinai Medical Centre, for instance, analysed electronic health records (EHRs) and genetic data using topological data analysis (TDA) to classify people with type 2 diabetes into three categories^23^. Insights on how individual patients will react to a medicine or a clinical trial were gleaned from the TDA’s patterns of clinical features and illness comorbidities. Patient identification using AI is at the cutting edge in areas such as the analysis of social media information. AI can sift through patient support groups’ worth of online discussions to see if there are any clusters of illness in any given area. This method may aid in the rapid identification of cohorts, which would aid businesses in the efficient planning of clinical trials. Once a target demographic has been determined, AI may assist with recruiting. The use of AI may streamline the hiring process and cut down on superfluous checks (as shown in Fig. 2).

## Applications

### Compiling information for distributed testing

One of the challenges of conducting decentralised clinical trials is managing the massive amounts of data that must be collected and analysed^24,25^. Patients must voluntarily and routinely provide their own participation data, as they are not present throughout the study. This may lead to problems with patient compliance and informational inaccuracies.

Medical research organisations and contract research organisations (CROs) may use AI to address these challenges in a number of ways. In this scenario, the intended objective is regular patient compliance, which may be achieved via the development of algorithms that analyse patient data and generate judgements.

The quality of the data submitted by patients may be evaluated by AI programmes before being accepted, which can be a great help to patients. An AI system, for instance, may examine a picture to determine whether it meets the criteria for a clinical study^26^. It may then provide the patient with advice on how to improve the quality of the photograph they took, such as how to adjust the lighting or the angle at which they took the picture. This reduces the number of incomplete or poorly constructed submissions, which in turn reduces the number of mistakes made when processing the data.

### Patient recruiting using data mining

Clinical trials and medication development rely heavily on the recruitment phase. Clinical trials fail if research organisations are unable to find enough participants quickly enough or if their recruiting efforts are ineffective. By sifting through population data, AI can help find the kind of people who would get the most out of taking part in clinical research^27^. By analysing the data and pinpointing the nations or areas where the illness is most common, an AI programme may also help choose the best sites for patient recruitment. In addition, it can scour medical records for upcoming trials. Finally, AI algorithms may analyse the available data on the illness and compare it to the characteristics of the clinical study to come up with eligibility criteria. They will be able to streamline these criteria and provide them to prospective members of the study.

### Biosimulation

Drug effectiveness and safety testing are important parts of clinical studies. Researchers may use biosimulation to conduct human tests digitally and measure the human reaction to the medicinal chemical before the medicine ever reaches human testing^28^. Biosimulation is the practise of simulating biological systems and processes on a computer using mathematical models. The simulation generates predictions based on models and data using AI and machine learning, which can then be used by researchers to understand more about the drug’s effects.

Biosimulation models use AI algorithms for pattern recognition in clinical trials and the analysis of drug-patient-demographic-trial parameter connections. These models allow researchers to explore questions about things like optimal dosing, medication interactions, and population-level efficacy. For instance, VeriSIM Life’s BIOiSIM platform uses AI and ML to mimic the effects of chemicals on both individual organs and whole bodily systems^29^.

### Methods of early disease diagnosis

Hospitals and medical research institutions are starting to develop disease-detection algorithms. Symptoms, medical histories, and diagnostic processes are studied and discussed in these programmes. The algorithm can then determine whether or not a patient is at risk for or experiencing the onset of a disease. Disease development may be slowed or symptoms alleviated by using detection algorithms to provide early care and therapy. CROs may use the algorithms to find and enrol patients at an earlier stage of the disease’s development, during the prodromal phase. IQVIA has created a data-driven illness detection programme that can assess a patient’s symptoms and characteristics, provide treatment recommendations (including clinical trials), and make expert referrals^30^.

## Difficulties with AI

Without a doubt, there will be hiccups and problems when introducing new technology. In the case of a sophisticated but still evolving and improving technology like AI, this is particularly true.

Integration of AI technology into clinical trials is difficult despite examples of its usefulness in population selection and enrolment. It is challenging to properly apply AI to create new sets of data due to the variety of forms and quality levels of medical record data. It would be great to have a single worldwide method for gathering and utilising data. Building a system to organise and store data is a critical part of developing AI. This will strengthen digital health solutions and advance technology.

AI also has to deal with the inherent bias in the data it uses. When an algorithm is trained using such datasets, it risks excluding large segments of the population that have been underrepresented in the past.

### Imperfect databases

The inherent bias in research datasets is one of AI’s biggest obstacles. Patients of European and Caucasian descent are overrepresented in medical studies and genetic databases^31,32^. Medical research suffers from a serious lack of diversity since it often treats able-bodied white people as the ‘default’. Since these groups have been historically underrepresented yet make up a significant fraction of the population, it follows that an AI trained on these datasets has insufficient knowledge of them. That leads to biased findings that may not apply to those groups or to patient pools that do not include those groups.

Although AI has the potential to revolutionise clinical trials, it still faces several obstacles. Faxed requests for patient records are still common in many clinical studies, with hospitals often responding with PDFs or photos (even photographs of handwritten notes)^33^. Unstructured data may likewise be created by using these techniques of communication. When a spreadsheet is faxed or converted to a read-only document (like a PDF), for instance, much of its original structure is lost. Researchers conducting clinical trials have a hard time collecting the precise information required to assess a patient’s eligibility using the current manual approach in place^34,35^.

### Cost-effectiveness and ease of access

New technologies often have price tags attached to their introduction. In the early stages of using new technologies, supply may be limited, which may drive up the cost of acquisition or rental. It may also be expensive to manufacture in bulk once it reaches the machinery market. In addition, many hospitals and CROs may not have access to this technology. Production and distribution of AI technology may be restricted to a select number of businesses.

## Role of ML in clinical trials

### Detection, acquisition, and maintenance of patients

It is difficult to select and recruit the best possible patients for clinical trials, despite the fact that many companies devote significant resources to participant management in an effort to reduce the likelihood of trial failure. Studies are often delayed, go over budget, or fail to yield usable data as a result of participant dropout and non-adherence. Nearly a third of all phase III studies fail because of enrolment issues, and it is estimated that 86% of all trials do not reach recruitment schedules. Clinical trials may be shortened, and the chance of failures not related to the medicine examined is reduced if these difficulties are addressed early on. Here are some of the ways in which ML may aid researchers in reducing error:

Analysis of patient population data to determine characteristics of individuals who are most likely to benefit from the proposed treatment or intervention (sometimes referred to informally as ‘responders’), hence improving the selection of particular patient groups for trials. Enhancing the processes of identifying and recruiting trial participants, for example, by using EHR data to match patients with studies^36,37^.

AI-powered voice assistants are already being used in many clinical trials for a variety of routine monitoring tasks, including notifying patients of upcoming appointments, keeping track of patients’ daily activities, facilitating better communication between sites and sponsors, and raising physicians’ levels of awareness^38,39^. The treatment of patients may be simplified and improved with the use of digital technologies since they are not limited by the capacity of the human intellect. Adopting ML techniques has been proven in simulation studies to improve treatment allocation and overall response rates for trial participants.

However, there are obstacles to utilising ML to identify more specific patient groups, particularly bias, which may distort and restrict ML results. For instance, enrolment may be unjustly skewed towards patient groups who have better access to healthcare and, therefore, a larger possibility of being picked based on their healthcare data if ideal patients are selectively identified using EHRs and other accessible data. Patients living in underserved, rural, or otherwise inaccessible places have fewer opportunities to participate in this research because they have less contact with the healthcare system overall. Although research with biased enrolment could fare better in terms of clinical trial success, its findings will not be generalisable to the patient population as a whole. In theory, this might lead to the approval of pharmaceuticals that are effective in treating a disease or condition in just a tiny subset of the target population.

It is possible that trial participants may feel their privacy is being violated if face recognition software or other forms of ML are used to keep track of participants and make sure they stick to the rules of the study^40^. Possible sources of bias include selecting patients who have access to and are familiar with the requisite ML monitoring technologies.

Women, people of colour, and patients from lower socioeconomic backgrounds have long been underrepresented in clinical trials, posing methodological and ethical challenges. Companies will need to include health equity considerations in their AI and ML systems to prevent these problems from being exacerbated. Some have proposed a ‘domain-forward approach’, in which domain experts (such as medical professionals) are brought into the algorithm development process to help fill in crucial context gaps that lead to inaccurate algorithmic decisions^41^. One way to make sure the real-world ramifications of the choices made by the algorithms are recognised is to include patients and carers in the development process. Companies interested in creating AI or ML algorithms for use in clinical trials should also establish procedures for regularly assessing and correcting any biases that may be present in such algorithms.

### Data management and collection

Data gathering and management are where AI and ML really shine in clinical research. ML may facilitate researchers’ data collection, processing, and management by enabling the use of digital health technology such as wearables, speech technologies, and computer vision for remote patient monitoring. Longitudinal and real-time biometric data collection made possible by these technologies may shed light on the lasting effects of medicines and treatment procedures in the real world.

The requirement for participants to go to study visits and have their data gathered in conventional ways may be avoided by employing alternative means of data collection, such as patient-generated health data through wearable devices. Facilitating the effective conduct and patient-centeredness of clinical trials via the use of novel digital biomarkers^42,43^.

The FDA must first approve new biomarkers before they can be widely used in ML processing of device data, and there has to be a deeper understanding of the overlap between previously approved clinical endpoints and patient-centred digital biomarkers^44–46^. It is also important to get the whole story on how people who use these devices feel about their data being shared and used. For both ethical and privacy considerations, researchers who want to make use of AI and ML-enabled devices must provide patients with explicit explanations of the risks and advantages of their data gathering. Participant recruitment and retention can suffer if worries about the technology being utilised are not addressed.

## Recent collaborations of industry with AI and clinical trials

### AstraZeneca

The collaboration between AstraZeneca and BenevolentAI to research therapies for CKD and IPF was announced in May 2019^9^. It began working with DeepMatter in December 2019 with the goal of increasing the efficiency of chemical synthesis via the use of AI-enabled digital technologies. In August of 2020, it formed a strategic alliance with Renalytix AI to advance the field of precision medicine for cardiovascular, renal, and metabolic disorders^47^.

### Takeda

Takeda has also partnered with AI companies to create new medicines. Takeda and Prometheus Biosciences joined together to research and develop new treatments for IBD^48^. Takeda and Prometheus Biosciences have teamed up to develop up to three targeted therapies and companion diagnostics by using the respective companies’ unique bioinformatics discovery platforms and cutting-edge ML methodologies. In addition, in January 2020, a fruitful collaboration was established with Recursion. Its partnership with Takeda in the field of rare illnesses has yielded the examination of preclinical and clinical molecules in more than 60 distinct indications and the identification of novel treatment options for a number of disorders, all in the space of less than 18 months. TAK-733 (REC-4881), a MEK inhibitor under clinical development for the treatment of a hereditary cancer syndrome and associated oncological conditions, was licenced to Recursion in May 2020^49^.

### BMS or Bristol-Myers Squibb

BMS and ReviveMed began working together in July 2020 to utilise their AI platform to investigate how cancer patients respond to and develop resistance to immunotherapies^50^. In October of 2020, insitro, a firm focused on accelerating medication discovery and development via the use of ML, became a partner^51^. The goal of the 5-year partnership is to find and create new medicines for the treatment of amyotrophic lateral sclerosis and frontotemporal disorders. BMS and Sensyne Health formed a partnership the same year to use ML to study the development of myeloproliferative neoplasms, a category of uncommon blood diseases^52^.

### Medidata

The solutions provided by Medidata AI may hasten medication development, lessen risks, save costs, and improve patient outcomes. Drug development may be sped up, lowered in cost, and improved in quality with the help of Medidata AI, which employs AI algorithms and ML to aid hospitals and CROs in executing clinical trials. Drug research and clinical trial efficiency are both areas where Medidata AI’s technologies are being employed by the pharmaceutical and biotech industries. Medidata AI’s products are utilised by academic institutions and government organisations to enhance clinical research and patient outcomes; for instance, Launch Therapeutics has chosen Medidata AI Intelligent Trials to speed up the preparation of clinical trials^53^. Intelligent Trials is a clinical trial analytics system from Medidata that uses AI to provide a competitive advantage in trial design and execution via the use of cross-industry real-time performance measurements, predictive models, and forecasting capabilities^54^.

### Saama

To complement its current array of tailored solutions and services, Saama, a supplier of AI-based and ML-based solutions that speed up clinical research and commercialisation, has released its unified platform of SaaS-based products. Clinical trial sponsors and CROs may save up to 90 min per query on query identification and generation time when utilising Saama’s cutting-edge technologies, as well as up to 50 min on data transformation time, 35 min on data input to analysis time, and more. Saama’s new platform utilises its tried-and-true AI and ML-enhanced solutions to streamline essential phases of clinical research and provide a bird’s-eye perspective of trial operations and patient status updates. There are many obstacles in today’s clinical research and development environment, such as the high cost of developing new treatments, the time and resource constraints involved in bringing more therapies to market, the ever-increasing volume and speed of patient data, and the talent gap that prevents the development of potentially life-saving treatments. Saama’s platform automates resource-intensive steps in clinical development with AI and big data analysis, allowing life science companies to increase productivity and better understand patient habits and reactions in real time. Accurately training AI models requires years and a massive amount of data. Saama contains over 90 models specifically designed for life sciences and trained on over 300 million data points, the result of over a decade of AI development. These models are integrated right into Saama, making it easy to roll out updates to the platform and its products as the platform grows and adapts to meet the demands of its users^55^.

### Other companies

Medication compliance in real-time research may be improved with the use of connected devices. Health (formerly Pillsy) released a mobile app that offers reminders, instructional material, dosage monitoring, and patient-reported data possibilities for doctors^56^. The startup, which has rebranded itself as a platform for remote patient monitoring, secured a $15 million Series A round in August of 2020.

Some methods concentrate on collecting biometric information. Using ML on real-time data, the AliveCor wearable electrocardiogram (EKG) gadget may identify irregular cardiac rhythms like atrial fibrillation (AF)^57^. The business began working with Medable in April of 2020 to facilitate remote clinical trials in cardiology. In November, AliveCor closed a $65 million Series E funding round.

## Regulatory

There is currently no standardised regulatory framework for the use of AI in medical research. However, a set of harmonised laws on AI was proposed by the European Commission (EC) as part of the Artificial Intelligence Act (AIA). It intends to ‘ensure that AI is safe, lawful, and in line with EU fundamental rights’ and ‘stimulate the uptake of trustworthy AI in the EU economy’^58^.

The AIA takes a precautionary approach. Systems that ‘pose significant risks to the health and safety or fundamental rights of persons’ and those that can ‘lead to biassed results and entail discriminatory results’ are examples of high-risk AI systems that must meet mandatory requirements for trustworthy AI and undergo a conformity assessment. Articles 30 and 43 of the AIA describe the conformity evaluation and focus on medical devices and *in vitro* diagnostic medical devices in particular^59^.

In addition, the AIA covers topics including restricted AI applications, provider and user responsibilities, transparency mandates, testing environments, expert labs, and penalties. The European Parliament is now reviewing the AIA after years of input iterations. In April 2022, the Committee on the Environment, Public Health, and Food Safety issued a position paper focusing on three issues: a lack of environmental protection, inadequate attention to users, and the need to handle both end users and the environment as risks.

Finally, the drug development process is spanned by the wide-ranging applications of AI-enabled technologies and ML in clinical research. The shown application cases demonstrate how AI-enabled technology and ML contribute to major advances in clinical research. This sector, like many others, is at the outset of a new journey, with its corresponding rules still in their infancy. The AIA is the EC’s first effort to regulate AI applications across sectors to guarantee respect for human rights. The future of clinical research and the advancement of AI-enabled technology are uncertain.

There has been a dramatic increase in the use of AI and ML across a wide variety of fields during the last decade. Interest in applying AI and ML to trial design and clinical trials to improve high failure rates is growing as a result of these factors: the continuous development of technology, access to ever more powerful computers, the increased availability of clinical and research data, and the rapid development of novel algorithms that analyse and utilise that data.

AI and ML have many potential real-world applications, including streamlining data management (e.g. automating data collection, monitoring data quality, and analysing large datasets) and reducing the risk of error in clinical trial participant management (e.g. cohort selection, patient identification and recruiting, participant retention). To use this technology to its full potential, however, a number of problems need to be solved. These include problems with the quality and availability of data, the openness of the development and validation processes, the possibility of bias in both the source data and the way the algorithm is used, and the lack of clear regulatory guidance from the government agencies involved.

## Limitations of AI in clinical trial

There has been a lot of buzz about using AI in healthcare and clinical trials recently. Although AI has the potential to revolutionise many facets of clinical research, it is essential that its limits and obstacles be understood. Key AI constraints in clinical trials have been explained below.

Without human judgement AI systems can sift through mountains of data and draw conclusions about what could happen next. However, they cannot take into account human faculties like common sense, intuition, and medical training. Multiple considerations, including patient history, comorbidities, and possible confounding variables, must typically be taken into account while making decisions in clinical trials. There is a risk that AI models could miss critical contextual subtleties and factors that might affect the result of a study.

Training data quality and variety have a significant impact on the accuracy and dependability of AI models. Complex, varied, and biased data from clinical trials are all possibilities. The AI model’s predictions and generalisability may be jeopardised if the training data is inadequate, biased, or otherwise unrepresentative. In addition, the lack of data on uncommon illnesses or disorders might impede the performance of AI models in clinical trial settings.

Complexities and ambiguities in regulatory frameworks are exacerbated when AI is used in clinical studies. Guidelines and standards for AI-driven healthcare solutions are still being developed by regulatory authorities like the FDA. It may be difficult to validate and understand the results of AI models in a way that satisfies regulatory requirements, since this usually requires extensive testing, validation, and explanation of the underlying algorithms. Privacy, informed consent, and data security are still issues of concern in the context of AI-driven clinical studies.

Ethical issues integrating AI into clinical studies brings up important ethical issues that must be considered. When AI algorithms are deployed, it might be difficult to get patients’ informed permission since they may not fully comprehend how the AI model works or how their data will be used. In order to earn patients’ confidence and maintain a high standard of ethical behaviour, it is essential that AI models be transparent and easy to understand. To avoid discrimination and unjust treatment, it is also important to address concerns about data privacy, data ownership, and the possibility of bias in AI algorithms.

It may be difficult and time-consuming to incorporate AI technology into pre-existing clinical trial procedures and systems. Many people, including scientists, doctors, patients, and government agencies, have a stake in clinical trials. Adopting AI calls for major adjustments to systems, procedures, and employee education. Individuals who are sceptical about or inexperienced with AI may be resistant to the integration process, and there is often a steep learning curve associated with adopting and maintaining AI systems.

Many AI systems, including deep learning neural networks, are termed ‘black box’ models due to the difficulty of understanding and explaining their inner workings. Since it is often unclear why an AI model arrived at a certain prediction or suggestion, this lack of interpretability might slow down mainstream adoption. Patient safety, regulatory compliance, and faith in the technology all depend on being able to explain and defend judgements made during clinical trials.

Concerns have been raised about the capacity of AI models trained on certain datasets to generalise and adapt to new data or new populations. AI models designed and trained on one group of patients or institutions may not perform as well when applied to various populations or situations, which is a common problem in clinical trials because of the large variety of individuals involved. AI has shown promise in clinical trials, but its lack of generalisability and transferability may restrict its use and scalability.

In conclusion, AI has the potential to dramatically improve clinical trials, but its limitations must be taken into account. To get through these barriers, researchers will need to work together and think critically about the ethical, regulatory, and practical difficulties they will face.

The advantages and limitations of AI in clinical trials have been tabulated in Table 1.

**Table 1:** Advantages and limitations of using artificial intelligence (AI) in clinical trials.

Advantages	Limitations
AI can transform key steps of clinical trial design, from study preparation to execution, leading to improved trial success rates	Requires significant investment in technology and infrastructure
Improve patient recruitment and protocol design, leading to higher chances of trial success	Requires specialized expertise in both clinical research and machine learning
Monitor patients and analyse data, leading to more accurate measurement and interpretation of results	Raises ethical concerns around data privacy and informed consent
Predict clinical outcomes, which is essential for precision medicine and can help eliminate statistical variability of general populations	Lack of empirical data validating the effectiveness of AI-based medications in planned clinical trials is the main obstacle to successful deployment
Allows healthcare professionals to better understand the patterns and needs of their patients through in-depth data analysis	In clinical trials, it must be verified how accurately the established AI algorithms solution works as compared to the clinical standards like sensitivity and specificity of diagnostic tests

## Conclusion

Although ML has much promise for enhancing the effectiveness and reliability of clinical research, many significant obstacles remain (some of which have already been touched on). Further guidance and standard-setting by the FDA and other regulatory agencies regarding transparency, oversight, and data quality in AI–ML processes will be necessary to decrease risks and discriminatory outcomes in clinical trials, in addition to the unresolved issues of inherent bias, informed consent, etc. that companies must address with their algorithms. Until then, enthusiasm for ML’s potential uses in clinical trials is likely to outstrip its actual utilisation.

Companies are always on the lookout for methods to improve the quality, cost, and safety of the clinical trial process in light of the many obstacles inherent in bringing a new medication or therapy to market. AI has the potential to speed trial cycles and patient outcomes by aggregating data in a manner that improves recruitment, adherence, and data analysis.

By releasing previously inaccessible, sophisticated insights, paving the way for automation, and hastening the whole clinical trial value chain, AI is poised to be the most revolutionary new technology in drug development. As AI develops and industry standards catch up, more advanced tools will become available to speed up the drug development process. These tools will help businesses recruit a wider range of people, keep them involved in the study longer, speed up the trials, save expenses, and increase the amount of data that can be reused. There is a lot of room for growth and development in the area of personalised medicine if AI is used. AI has a long way to go before it is widely accepted by the clinical trial community, though.

In conclusion, AI has the ability to revolutionise every aspect of running clinical trials, from finding participants to analysing the results. CROs and medical institutions may improve the efficiency, economy, and timeliness of drug development by using AI algorithms and ML. However, there are obstacles that must be overcome, such as poor data quality, ethical concerns, and a lack of data governance. We should anticipate seeing revolutionary changes in drug development and clinical research thanks to the use of AI in clinical trials in the near future.

## Ethical approval

Not applicable.

## Sources of funding

No funding was received.

## Author contribution

H.C.: conceptualization, data curation, writing – original draft preparation, reviewing, and editing; A.: data curation, writing – original draft preparation, reviewing, and editing; D.K.S.: writing – original draft preparation, reviewing, and editing, visualisation, and supervision; K.M.: writing revision, editing, and figure preparation; P.: writing revision, editing, and supervision; K.D.: data curation, writing – original draft preparation, reviewing, and editing; T.B.E.: writing – reviewing and editing, visualisation, and supervision.

## Conflicts of interest disclosure

Authors declare that they have no conflicts of interest.

## Research registration unique identifying number (UIN)

1. Name of the registry: not applicable.
2. Unique Identifying number or registration ID: not applicable.
3. Hyperlink to your specific registration (must be publicly accessible and will be checked): not applicable.

## Guarantor

Talha Bin Emran, PhD, Associate Professor, Department of Pharmacy, BGC Trust University Bangladesh, Chittagong 4381, Bangladesh; Tel: +88 030 3356193, fax: +88 031 2550224, Cell: +88 01819942214; https://orcid.org/0000-0003-3188-2272.

## Data availability statement

The data in this correspondence article is not sensitive in nature and is accessible in the public domain. The data is therefore available and not of a confidential nature.

## Provenance and peer review

Not commissioned, internally peer-reviewed.
